# (2,4-Dihy­droxy-6-meth­oxy­phen­yl)(3,5-dihy­droxy­phen­yl)methanone monohydrate

**DOI:** 10.1107/S1600536811037913

**Published:** 2011-09-30

**Authors:** Jamila Nargis, Keng-Chong Wong, Melati Khairuddin, Suchada Chantrapromma, Hoong-Kun Fun

**Affiliations:** a School of Chemical Sciences, Universiti Sains Malaysia, 11800 USM, Penang, Malaysia; b Crystal Materials Research Unit, Department of Chemistry, Faculty of Science, Prince of Songkla University, Hat-Yai, Songkhla 90112, Thailand; c X-ray Crystallography Unit, School of Physics, Universiti Sains Malaysia, 11800 USM, Penang, Malaysia

## Abstract

The title benzophenone compound, C_14_H_12_O_6_·H_2_O, was isolated from the bark of *Garcinia hombroniana* Pierre (Guttiferae). The mol­ecule is twisted, the dihedral angle between the two benzene rings being 59.13 (7)°. The meth­oxy group is approximately coplanar with the attached benzene ring, with a C—O—C—C torsion angle of 1.91 (18)°. The water mol­ecule is disordered over two positions in a 0.555 (19):0.445 (19) ratio. An intra­molecular O—H⋯O hydrogen bond generates an *S*(6) ring motif. The crystal structure is stabilized by inter­molecular O—H⋯O hydrogen bonds. These inter­actions link the mol­ecules into sheets parallel to the *ac* plane. The sheets are stacked along the *b* axis by π–π inter­actions, with centroid–centroid distances of 3.6219 (7) Å. A weak O—H⋯π inter­action was also noted.

## Related literature

For details of hydrogen-bond motifs, see: Bernstein *et al.* (1995 ▶). For background to benzophenones and their bioactivity, see: Khanum *et al.* (2009 ▶); Pereira *et al.* (2010 ▶); Tzanova *et al.* (2009 ▶). For background to Guttiferae plants, see: Jayaprakasha *et al.* (2006 ▶); Mahabusarakum *et al.* (1983 ▶); Ngoupayo *et al.* (2009 ▶); Pereira *et al.* (2010 ▶); Phongpaichit *et al.* (1994 ▶); Smitinand (2001 ▶); Zadernowski *et al.* (2009 ▶); Zhang *et al.* (2010 ▶). For related structures, see: Betz *et al.* (2011 ▶); Li *et al.* (2010 ▶). For stability of the temperature controller used in the data collection, see Cosier & Glazer (1986 ▶).
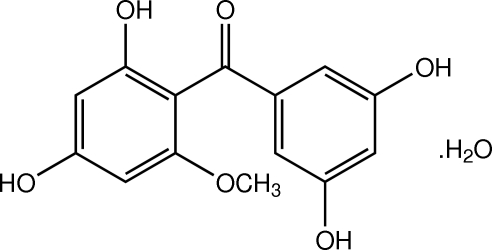


## Experimental

### 

#### Crystal data

C_14_H_12_O_6_·H_2_O
*M* *_r_* = 294.25
Triclinic, 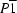

*a* = 7.7087 (1) Å
*b* = 8.4050 (1) Å
*c* = 11.2380 (1) Å
α = 82.401 (1)°
β = 75.570 (1)°
γ = 67.842 (1)°
*V* = 652.49 (1) Å^3^
*Z* = 2
Mo *K*α radiation
μ = 0.12 mm^−1^
*T* = 100 K
0.42 × 0.33 × 0.10 mm

#### Data collection

Bruker APEXII CCD area-detector diffractometer
Absorption correction: multi-scan (*SADABS*; Bruker, 2005 ▶) *T*_min_ = 0.951, *T*_max_ = 0.988
19157 measured reflections
4696 independent reflections
4220 reflections with *I* > 2σ(*I*)
*R*_int_ = 0.023

#### Refinement

*R*[*F* ^2^ > 2σ(*F* ^2^)] = 0.052
*wR*(*F* ^2^) = 0.131
*S* = 1.17
4696 reflections
217 parameters
H atoms treated by a mixture of independent and constrained refinement
Δρ_max_ = 0.51 e Å^−3^
Δρ_min_ = −0.31 e Å^−3^

### 

Data collection: *APEX2* (Bruker, 2005 ▶); cell refinement: *SAINT* (Bruker, 2005 ▶); data reduction: *SAINT*; program(s) used to solve structure: *SHELXTL* (Sheldrick, 2008 ▶); program(s) used to refine structure: *SHELXTL*; molecular graphics: *SHELXTL*; software used to prepare material for publication: *SHELXTL* and *PLATON* (Spek, 2009 ▶).

## Supplementary Material

Crystal structure: contains datablock(s) global, I. DOI: 10.1107/S1600536811037913/tk2790sup1.cif

Structure factors: contains datablock(s) I. DOI: 10.1107/S1600536811037913/tk2790Isup2.hkl

Supplementary material file. DOI: 10.1107/S1600536811037913/tk2790Isup3.cml

Additional supplementary materials:  crystallographic information; 3D view; checkCIF report

## Figures and Tables

**Table 1:** Hydrogen-bond geometry (Å, °) *Cg*2 is the centroid of the C8–C13 ring.

*D*—H⋯*A*	*D*—H	H⋯*A*	*D*⋯*A*	*D*—H⋯*A*
O2—H1*O*2⋯O1	0.89 (3)	1.72 (3)	2.5453 (14)	152 (3)
O2—H1*O*2⋯O1^i^	0.89 (3)	2.45 (3)	2.9554 (16)	117 (3)
O3—H1*O*3⋯O5^ii^	0.85 (2)	1.90 (2)	2.7440 (15)	177 (3)
O5—H1*O*5⋯O1*W*^iii^	0.83 (3)	1.76 (3)	2.574 (7)	166 (3)
O6—H1*O*6⋯O2^iv^	0.79 (3)	1.98 (3)	2.7220 (15)	157 (3)
O1*W*—H1*W*1⋯O6^ii^	0.89	1.91	2.746 (8)	156
C14—H14*C*⋯O1^v^	0.98	2.55	3.4507 (19)	152
O1*WX*—H2*WX*⋯*Cg*2^vi^	0.86	2.89	3.402 (7)	120

Symmetry codes: (i) 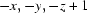; (ii) 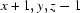; (iii) 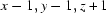; (iv) 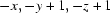; (v) 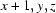; (vi) 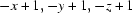.

**Fig. 1.**
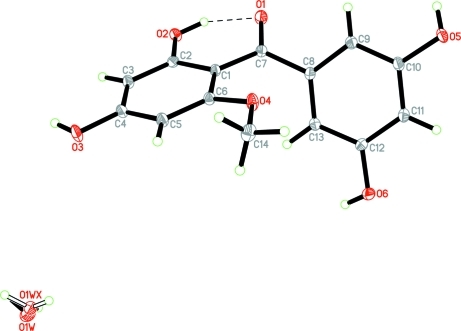
The molecular structure of the title compound, with 50% probability displacement ellipsoids and the atom-numbering scheme. Open bonds show the minor component of the disordered water molecule. The hydrogen bond is shown as a dashed line.

**Fig. 2.**
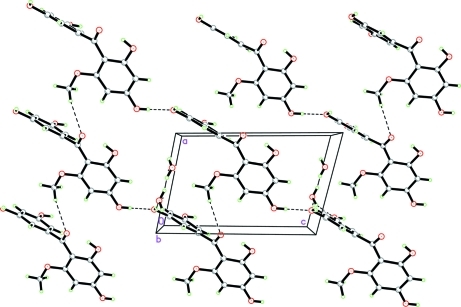
The crystal packing of the title compound with only the major component of the water molecule shown, viewed down the b axis, showing sheets parallel to the ac plane. Hydrogen bonds are shown as dashed lines.

## supplementary crystallographic information

### Comment

*Garcinia* is traditionally used for the treatment of abdominal pain, dysentery, diarrhea, suppuration, infected wounds, leucorrhoea, chronic ulcers and gonorrhea (Jayaprakasha *et al.*, 2006). Most *Garcinia* species are trees but some are shrubs or treelets and are rich sources of bioactive compounds including xanthones, flavonoids, benzophenones, lactones and phenolic acids (Mahabusarakum *et al.*, 1983; Ngoupayo *et al.*, 2009; Pereira *et al.*, 2010; Phongpaichit *et al.*, 1994; Zadernowski *et al.*, 2009; Zhang *et al.*, 2010). *Garcinia hombroniana* Pierre (Guttiferae), commonly called seashore mangosteen or pokok bruas (Malay), is indigenous in Malaysia and widely distributed in the southern part of Thailand where it is known as "wa" (Smitinand, 2001). During the course of our investigation of *G. hombroniana*, we have isolated the title benzophenone compound (I) from the ethyl acetate extract of the bark. Benzophenones have been found to possess antioxidant (Tzanova *et al.*, 2009), anti-inflammatory (Khanum *et al.*, 2009) and leishmanicidal (Pereira *et al.*, 2010) activities.

The molecule of the title benzophenone which crystallized as a monohydrate (Fig. 1) is twisted with the dihedral angle between the 2,4-dihydroxy-6-methoxyphenyl and 3,5-dihydroxyphenyl rings being 59.13 (7)°, whereas the triangular C_aryl_-C(═O)-C_aryl_ fragment (C1/C7/C8/O1) makes dihedral angles of 25.16 (7) and 43.44 (8)° with the 2,4-dihydroxy-6-methoxyphenyl and 3,5-dihydroxyphenyl rings, respectively. The two hydroxy groups and the methoxy groups of the 2,4-dihydroxy-6-methoxyphenyl residue are co-planar with the benzene ring with a *r.m.s.* of 0.0459 (1) Å for the ten non-H atoms and the torsion angle C14–O1–C6–C5 = 1.91 (18)°. The two hydroxy groups of the 3,5-dihydroxyphenyl are also co-planar with the *r.m.s.* of 0.0083 (1) Å for the eight non-H atoms. An intramolecular O2—H1O2···O1 hydrogen bond generates a S(6) ring motif (Bernstein *et al.*, 1995) (Fig. 1 and Table 1). The bond distances are comparable with the related structures (Betz *et al.*, 2011; Li *et al.*, 2010). The water molecule is disordered over two positions in a 0.555 (19):0.445 (19) ratio.

The crystal is stabilized by intermolecular O—H···O hydrogen bonds. These interactions link the molecules into sheets parallel to the *ac* plane (Fig. 2). These sheets are stacked along the *b* axis by π···π interaction with the Cg_1_···Cg_1_ distance of 3.6219 (7) Å (symmetry code 1-x, -y, 1-z) where Cg_1_ is the centroid of the C1–C6 benzene ring. A weak O—H···π interaction was also observed (Table 1).

### Experimental

The air-dried and ground bark (5.2 kg) of *G. hombroniana* was extracted (Soxhlet) successively with n-hexane, dichloromethane, ethyl acetate and methanol. The ethyl acetate extract (18 g) was subjected to silica gel column chromatography (CC) using hexane-chloroform-ethyl acetate-methanol gradient, affording 40 fractions (FE1- FE40). Fractions FE23-FE29 were combined and fractionated using silica gel CC with chloroform-acetone gradient which afforded another fraction FEb. The title compound (I) was isolated from the fraction FEb in the form of shiny yellow crystals by silica gel CC with chloroform-acetone (3:2 v/v) as eluting solvent. The melting point determined was 516-519 K.

### Refinement

Hydroxy H atoms were located from a Fourier difference map and isotropically refined. The C-bound H atoms were placed in calculated positions with d(C—H) = 0.95 Å for aromatic and 0.98 Å for CH_3_ atoms. The *U*_iso_ values were constrained to be 1.5*U*_eq_ of the carrier atom for O and methyl H atoms, and 1.2*U*_eq_ for the remaining H atoms. A rotating group model was used for the methyl groups. The water molecule was found to be disordered over two sites in a 0.555 (19): 0.445 (19) occupancy ratio (from refinement). The water-bound H1W1, H2W1, H1WX and H2WX atoms were located from a difference Fourier map and fixed in these positions with *U*_iso_(H) = 1.5 *U*_eq_(O)

### Crystal data

**Table:**

C_14_H_12_O_6_·H_2_O	*Z* = 2
*M**_r_* = 294.25	*F*(000) = 308
Triclinic, *P*1	*D*_x_ = 1.498 Mg m^−^^3^
Hall symbol: -P 1	Melting point = 516–519 K
*a* = 7.7087 (1) Å	Mo *K*α radiation, λ = 0.71073 Å
*b* = 8.4050 (1) Å	Cell parameters from 4696 reflections
*c* = 11.2380 (1) Å	θ = 1.9–32.5°
α = 82.401 (1)°	µ = 0.12 mm^−^^1^
β = 75.570 (1)°	*T* = 100 K
γ = 67.842 (1)°	Plate, yellow
*V* = 652.49 (1) Å^3^	0.42 × 0.33 × 0.10 mm

### Data collection

**Table:**

Bruker APEXII CCD area-detector diffractometer	4696 independent reflections
Radiation source: sealed tube	4220 reflections with *I* > 2σ(*I*)
graphite	*R*_int_ = 0.023
φ and ω scans	θ_max_ = 32.5°, θ_min_ = 1.9°
Absorption correction: multi-scan (*SADABS*; Bruker, 2005)	*h* = −11→11
*T*_min_ = 0.951, *T*_max_ = 0.988	*k* = −12→12
19157 measured reflections	*l* = −16→16

### Refinement

**Table:**

Refinement on *F*^2^	Primary atom site location: structure-invariant direct methods
Least-squares matrix: full	Secondary atom site location: difference Fourier map
*R*[*F*^2^ > 2σ(*F*^2^)] = 0.052	Hydrogen site location: inferred from neighbouring sites
*wR*(*F*^2^) = 0.131	H atoms treated by a mixture of independent and constrained refinement
*S* = 1.17	*w* = 1/[σ^2^(*F*_o_^2^) + (0.038*P*)^2^ + 0.633*P*] where *P* = (*F*_o_^2^ + 2*F*_c_^2^)/3
4696 reflections	(Δ/σ)_max_ = 0.001
217 parameters	Δρ_max_ = 0.51 e Å^−^^3^
0 restraints	Δρ_min_ = −0.31 e Å^−^^3^

### Special details

**Table:**

Experimental. The crystal was placed in the cold stream of an Oxford Cryosystems Cobra open-flow nitrogen cryostat (Cosier & Glazer, 1986) operating at 120.0 (1) K.
Geometry. All esds (except the esd in the dihedral angle between two l.s. planes) are estimated using the full covariance matrix. The cell esds are taken into account individually in the estimation of esds in distances, angles and torsion angles; correlations between esds in cell parameters are only used when they are defined by crystal symmetry. An approximate (isotropic) treatment of cell esds is used for estimating esds involving l.s. planes.
Refinement. Refinement of F^2^ against ALL reflections. The weighted R-factor wR and goodness of fit S are based on F^2^, conventional R-factors R are based on F, with F set to zero for negative F^2^. The threshold expression of F^2^ > 2sigma(F^2^) is used only for calculating R-factors(gt) etc. and is not relevant to the choice of reflections for refinement. R-factors based on F^2^ are statistically about twice as large as those based on F, and R- factors based on ALL data will be even larger.

### Fractional atomic coordinates and isotropic or equivalent isotropic displacement parameters (Å^2^)

**Table:**

	*x*	*y*	*z*	*U*_iso_*/*U*_eq_	Occ. (<1)
O1	0.02024 (14)	0.10822 (14)	0.58801 (9)	0.01558 (19)	
O2	0.18999 (14)	0.13186 (13)	0.36467 (9)	0.01292 (18)	
H1O2	0.114 (4)	0.106 (4)	0.432 (3)	0.044 (7)*	
O3	0.74976 (15)	0.27372 (15)	0.28766 (10)	0.0168 (2)	
H1O3	0.757 (4)	0.250 (3)	0.215 (2)	0.032 (6)*	
O4	0.40238 (14)	0.24500 (14)	0.69771 (9)	0.01396 (19)	
O5	−0.23219 (17)	0.21024 (15)	1.05072 (9)	0.0191 (2)	
H1O5	−0.247 (4)	0.123 (3)	1.035 (2)	0.036 (7)*	
O6	−0.12705 (15)	0.70856 (13)	0.85592 (10)	0.01557 (19)	
H1O6	−0.114 (4)	0.751 (4)	0.789 (3)	0.040 (7)*	
C1	0.27991 (17)	0.20544 (16)	0.53565 (11)	0.0104 (2)	
C2	0.31206 (17)	0.17912 (16)	0.40884 (11)	0.0106 (2)	
C3	0.46478 (18)	0.20447 (17)	0.32314 (12)	0.0125 (2)	
H3A	0.4796	0.1921	0.2380	0.015*	
C4	0.59587 (18)	0.24845 (17)	0.36499 (12)	0.0121 (2)	
C5	0.57783 (18)	0.26523 (17)	0.49021 (12)	0.0128 (2)	
H5A	0.6706	0.2921	0.5172	0.015*	
C6	0.42273 (17)	0.24217 (16)	0.57445 (11)	0.0110 (2)	
C7	0.10655 (17)	0.19008 (16)	0.61790 (12)	0.0110 (2)	
C8	0.01674 (17)	0.28193 (16)	0.73582 (11)	0.0107 (2)	
C9	−0.06148 (18)	0.19737 (17)	0.83798 (12)	0.0126 (2)	
H9A	−0.0505	0.0816	0.8341	0.015*	
C10	−0.15600 (19)	0.28606 (17)	0.94574 (12)	0.0131 (2)	
C11	−0.17726 (19)	0.45713 (17)	0.95104 (12)	0.0137 (2)	
H11A	−0.2429	0.5169	1.0247	0.016*	
C12	−0.10135 (18)	0.53944 (16)	0.84732 (12)	0.0117 (2)	
C13	−0.00183 (18)	0.45267 (17)	0.73944 (12)	0.0120 (2)	
H13A	0.0524	0.5089	0.6696	0.014*	
C14	0.54604 (19)	0.27856 (19)	0.73956 (13)	0.0159 (2)	
H14A	0.5188	0.2715	0.8297	0.024*	
H14B	0.5450	0.3939	0.7101	0.024*	
H14C	0.6725	0.1932	0.7076	0.024*	
O1W	0.6637 (7)	0.9646 (10)	0.0189 (7)	0.0211 (11)	0.555 (19)
H1W1	0.7461	0.8666	−0.0151	0.032*	0.555 (19)
H2W1	0.5642	0.9957	−0.0002	0.032*	0.555 (19)
O1WX	0.6960 (8)	0.9169 (9)	0.0518 (6)	0.0125 (9)	0.445 (19)
H1WX	0.6896	0.8759	0.1225	0.019*	0.445 (19)
H2WX	0.7964	0.8353	0.0173	0.019*	0.445 (19)

### Atomic displacement parameters (Å^2^)

**Table:**

	*U*^11^	*U*^22^	*U*^33^	*U*^12^	*U*^13^	*U*^23^
O1	0.0153 (4)	0.0209 (5)	0.0142 (4)	−0.0110 (4)	−0.0001 (3)	−0.0048 (4)
O2	0.0146 (4)	0.0173 (4)	0.0104 (4)	−0.0096 (4)	−0.0028 (3)	−0.0006 (3)
O3	0.0162 (4)	0.0256 (5)	0.0107 (4)	−0.0129 (4)	0.0013 (3)	0.0001 (4)
O4	0.0120 (4)	0.0231 (5)	0.0088 (4)	−0.0084 (4)	−0.0019 (3)	−0.0021 (3)
O5	0.0306 (6)	0.0212 (5)	0.0093 (4)	−0.0173 (4)	0.0021 (4)	−0.0008 (4)
O6	0.0230 (5)	0.0115 (4)	0.0118 (4)	−0.0067 (4)	−0.0022 (4)	−0.0006 (3)
C1	0.0097 (5)	0.0119 (5)	0.0097 (5)	−0.0046 (4)	−0.0009 (4)	−0.0013 (4)
C2	0.0112 (5)	0.0110 (5)	0.0102 (5)	−0.0041 (4)	−0.0029 (4)	−0.0013 (4)
C3	0.0134 (5)	0.0154 (5)	0.0096 (5)	−0.0068 (4)	−0.0012 (4)	−0.0005 (4)
C4	0.0114 (5)	0.0142 (5)	0.0110 (5)	−0.0063 (4)	−0.0007 (4)	0.0002 (4)
C5	0.0122 (5)	0.0161 (5)	0.0113 (5)	−0.0071 (4)	−0.0015 (4)	−0.0011 (4)
C6	0.0110 (5)	0.0131 (5)	0.0095 (5)	−0.0050 (4)	−0.0020 (4)	−0.0013 (4)
C7	0.0103 (5)	0.0121 (5)	0.0104 (5)	−0.0041 (4)	−0.0017 (4)	−0.0013 (4)
C8	0.0098 (5)	0.0133 (5)	0.0091 (5)	−0.0045 (4)	−0.0015 (4)	−0.0014 (4)
C9	0.0132 (5)	0.0137 (5)	0.0117 (5)	−0.0062 (4)	−0.0011 (4)	−0.0015 (4)
C10	0.0148 (5)	0.0166 (6)	0.0093 (5)	−0.0082 (5)	−0.0014 (4)	0.0002 (4)
C11	0.0159 (5)	0.0156 (6)	0.0098 (5)	−0.0066 (5)	−0.0006 (4)	−0.0019 (4)
C12	0.0122 (5)	0.0117 (5)	0.0114 (5)	−0.0038 (4)	−0.0029 (4)	−0.0017 (4)
C13	0.0124 (5)	0.0129 (5)	0.0105 (5)	−0.0053 (4)	−0.0008 (4)	−0.0008 (4)
C14	0.0139 (5)	0.0225 (6)	0.0136 (6)	−0.0070 (5)	−0.0043 (4)	−0.0046 (5)
O1W	0.0180 (11)	0.0201 (19)	0.026 (2)	−0.0063 (12)	−0.0030 (12)	−0.0079 (18)
O1WX	0.0132 (13)	0.0121 (16)	0.0115 (15)	−0.0032 (12)	−0.0041 (10)	0.0009 (12)

### Geometric parameters (Å, °)

**Table:**

O1—C7	1.2437 (16)	C5—H5A	0.9500
O2—C2	1.3626 (15)	C7—C8	1.4972 (18)
O2—H1O2	0.89 (3)	C8—C13	1.3925 (18)
O3—C4	1.3546 (15)	C8—C9	1.3961 (17)
O3—H1O3	0.85 (3)	C9—C10	1.3937 (18)
O4—C6	1.3570 (15)	C9—H9A	0.9500
O4—C14	1.4344 (16)	C10—C11	1.3920 (19)
O5—C10	1.3715 (16)	C11—C12	1.3900 (18)
O5—H1O5	0.83 (3)	C11—H11A	0.9500
O6—C12	1.3724 (16)	C12—C13	1.3923 (17)
O6—H1O6	0.79 (3)	C13—H13A	0.9500
C1—C2	1.4168 (17)	C14—H14A	0.9800
C1—C6	1.4244 (17)	C14—H14B	0.9800
C1—C7	1.4628 (17)	C14—H14C	0.9800
C2—C3	1.3888 (17)	O1W—H1W1	0.8916
C3—C4	1.3942 (18)	O1W—H2W1	0.7867
C3—H3A	0.9500	O1WX—H1WX	0.8209
C4—C5	1.3998 (18)	O1WX—H2WX	0.8602
C5—C6	1.3876 (17)		
			
C2—O2—H1O2	104.3 (18)	C13—C8—C9	121.23 (12)
C4—O3—H1O3	109.9 (16)	C13—C8—C7	120.11 (11)
C6—O4—C14	117.37 (10)	C9—C8—C7	118.45 (11)
C10—O5—H1O5	110.8 (18)	C10—C9—C8	118.71 (12)
C12—O6—H1O6	109 (2)	C10—C9—H9A	120.6
C2—C1—C6	116.96 (11)	C8—C9—H9A	120.6
C2—C1—C7	118.58 (11)	O5—C10—C11	116.99 (12)
C6—C1—C7	124.45 (11)	O5—C10—C9	122.05 (12)
O2—C2—C3	116.67 (11)	C11—C10—C9	120.96 (12)
O2—C2—C1	121.07 (11)	C12—C11—C10	119.21 (12)
C3—C2—C1	122.23 (11)	C12—C11—H11A	120.4
C2—C3—C4	118.45 (12)	C10—C11—H11A	120.4
C2—C3—H3A	120.8	O6—C12—C11	117.41 (11)
C4—C3—H3A	120.8	O6—C12—C13	121.52 (12)
O3—C4—C3	122.18 (12)	C11—C12—C13	121.06 (12)
O3—C4—C5	116.18 (11)	C12—C13—C8	118.81 (12)
C3—C4—C5	121.63 (12)	C12—C13—H13A	120.6
C6—C5—C4	119.19 (12)	C8—C13—H13A	120.6
C6—C5—H5A	120.4	O4—C14—H14A	109.5
C4—C5—H5A	120.4	O4—C14—H14B	109.5
O4—C6—C5	122.54 (11)	H14A—C14—H14B	109.5
O4—C6—C1	116.13 (11)	O4—C14—H14C	109.5
C5—C6—C1	121.26 (12)	H14A—C14—H14C	109.5
O1—C7—C1	120.63 (11)	H14B—C14—H14C	109.5
O1—C7—C8	117.04 (11)	H1W1—O1W—H2W1	112.7
C1—C7—C8	122.17 (11)	H1WX—O1WX—H2WX	97.6
			
C6—C1—C2—O2	175.50 (11)	C6—C1—C7—O1	−157.57 (13)
C7—C1—C2—O2	−3.32 (18)	C2—C1—C7—C8	−154.00 (12)
C6—C1—C2—C3	−6.35 (19)	C6—C1—C7—C8	27.27 (19)
C7—C1—C2—C3	174.83 (12)	O1—C7—C8—C13	−132.18 (13)
O2—C2—C3—C4	−178.32 (11)	C1—C7—C8—C13	43.14 (18)
C1—C2—C3—C4	3.46 (19)	O1—C7—C8—C9	42.51 (17)
C2—C3—C4—O3	179.32 (12)	C1—C7—C8—C9	−142.18 (13)
C2—C3—C4—C5	0.8 (2)	C13—C8—C9—C10	−1.36 (19)
O3—C4—C5—C6	179.57 (12)	C7—C8—C9—C10	−175.99 (11)
C3—C4—C5—C6	−1.8 (2)	C8—C9—C10—O5	−178.31 (12)
C14—O4—C6—C5	1.91 (18)	C8—C9—C10—C11	1.8 (2)
C14—O4—C6—C1	178.89 (11)	O5—C10—C11—C12	179.54 (12)
C4—C5—C6—O4	175.49 (12)	C9—C10—C11—C12	−0.5 (2)
C4—C5—C6—C1	−1.3 (2)	C10—C11—C12—O6	179.35 (12)
C2—C1—C6—O4	−171.78 (11)	C10—C11—C12—C13	−1.2 (2)
C7—C1—C6—O4	6.96 (19)	O6—C12—C13—C8	−178.96 (12)
C2—C1—C6—C5	5.23 (19)	C11—C12—C13—C8	1.58 (19)
C7—C1—C6—C5	−176.02 (12)	C9—C8—C13—C12	−0.30 (19)
C2—C1—C7—O1	21.15 (19)	C7—C8—C13—C12	174.24 (11)

### Hydrogen-bond geometry (Å, °)

**Table:**

*Cg*2 is the centroid of the C8–C13 ring.

**Table:**

*D*—H···*A*	*D*—H	H···*A*	*D*···*A*	*D*—H···*A*
O2—H1O2···O1	0.89 (3)	1.72 (3)	2.5453 (14)	152 (3)
O2—H1O2···O1^i^	0.89 (3)	2.45 (3)	2.9554 (16)	117 (3)
O3—H1O3···O5^ii^	0.85 (2)	1.90 (2)	2.7440 (15)	177 (3)
O5—H1O5···O1W^iii^	0.83 (3)	1.76 (3)	2.574 (7)	166 (3)
O6—H1O6···O2^iv^	0.79 (3)	1.98 (3)	2.7220 (15)	157 (3)
O1W—H1W1···O6^ii^	0.89	1.91	2.746 (8)	156
C14—H14C···O1^v^	0.98	2.55	3.4507 (19)	152
O1WX—H2WX···Cg2^vi^	0.86	2.89	3.402 (7)	120

Symmetry codes:  (i) −*x*, −*y*, −*z*+1; (ii) *x*+1, *y*, *z*−1; (iii) *x*−1, *y*−1, *z*+1; (iv) −*x*, −*y*+1, −*z*+1; (v) *x*+1, *y*, *z*; (vi) −*x*+1, −*y*+1, −*z*+1.
